# The potency of mitochondria enlargement for mitochondria-mediated terpenoid production in yeast

**DOI:** 10.1007/s00253-023-12922-5

**Published:** 2024-01-13

**Authors:** So Yanagibashi, Takahiro Bamba, Takayoshi Kirisako, Akihiko Kondo, Tomohisa Hasunuma

**Affiliations:** 1 Graduate School of Science, Technology and Innovation, Kobe University, 1-1 Rokkodai, Nada, Kobe, 657-8501 Japan; 2 Kirin Central Research Institute, Kirin Holdings Company, Ltd., 26-1-12-12 Muraoka-Higashi 2-Chome, Fujisawa, Kanagawa 251-8555 Japan; 3 Engineering Biology Research Center, Kobe University, 1-1 Rokkodai, Nada, Kobe, 6578501 Japan; 4 RIKEN Center for Sustainable Resource Science, 1-7-22 Suehiro-Cho, Tsurumi-Ku, Yokohama, Kanagawa 230-0045 Japan

**Keywords:** Terpenoid, Mitochondria, Yeast, Mevalonate pathway, Squalene, β-carotene

## Abstract

**Abstract:**

Terpenoids are widely used in the food, beverage, cosmetics, and pharmaceutical industries. Microorganisms have been extensively studied for terpenoid production. In yeast, the introduction of the mevalonate (MVA) pathway in organelles in addition to the augmentation of its own MVA pathway have been challenging. Introduction of the MVA pathway into mitochondria is considered a promising approach for terpenoid production because acetyl-CoA, the starting molecule of the MVA pathway, is abundant in mitochondria. However, mitochondria comprise only a small percentage of the entire cell. Therefore, we hypothesized that increasing the total mitochondrial volume per cell would increase terpenoid production. First, we ascertained that the amounts of isopentenyl diphosphate (IPP) and dimethylallyl diphosphate (DMAPP), the final molecules of the MVA pathway, were 15-fold higher of the strain expressing the MVA pathway in mitochondria than in the wild-type yeast strain. Second, we found that different deletion mutants induced different mitochondrial volumes by measuring the mitochondrial volume in various deletion mutants affecting mitochondrial morphology; for example,Δ*mdm32* increased mitochondrial volume, and Δ*fzo1* decreased it. Finally, the effects of mitochondrial volume on amounts of IPP/DMAPP and terpenoids (squalene or β-carotene) were investigated using mutants harboring large or small mitochondria expressing the MVA pathway in mitochondria. Amounts of IPP/DMAPP and terpenoids (squalene or β-carotene) increased when the mitochondrial volume expanded. Introducing the MVA pathway into mitochondria for terpenoid production in yeast may become more attractive by enlarging the mitochondrial volume.

**Key points:**

*• IPP/DMAPP content increased in the strain expressing the MVA pathway in mitochondria*

*• IPP/DMAPP and terpenoid contents are positively correlated with mitochondrial volume*

*• Enlarging the mitochondria may improve mitochondria-mediated terpenoid production*

**Supplementary Information:**

The online version contains supplementary material available at 10.1007/s00253-023-12922-5.

## Introduction

Terpenoids are a large group of natural compounds constructed from isoprene units (C5). Many different terpenoids are used in the food, beverage, cosmetic, and pharmaceutical industries (Tetali 2019). The current industrial supply of terpenoids relies mainly on chemical synthesis or extraction from plants. The chemical synthesis of terpenoids harboring complex structures has been actively researched (Phil 2018). However, the multi-stereo center in the complex terpenoids requires diastereoselective synthesis, resulting in multi-step total synthesis with high costs and low yield (Santosh et al. 2020; Le et al. 2022). Therefore, the chemical synthesis of terpenoids harboring complex structures is expensive and requires fine-controlled and complex processes. Plant extraction has been also used to supply terpenoids; however, it is limited by the presence of various compounds harboring similar physicochemical properties in plants and the difficulty of separating these compounds (Rodger et al. 2011). In addition, natural plant supplies are limited owing to their finite amount (Nur and Henrik 2017). Therefore, the engineering of microorganisms to produce terpenoids has emerged as a promising approach.

Research on terpenoid production using microorganisms has rapidly increased in the twenty-first century. *Saccharomyces cerevisiae* and *Escherichia coli* have been used to produce plant terpenoids by heterologous expression of their genes because they have advantages for production, such as the presence of a native terpenoid synthetic pathway and facile genetic manipulation (Navale et al. 2021; Sun et al. 2020). *Saccharomyces cerevisiae* offers several advantages for terpenoid production, including a high sugar catabolic rate, a relatively fast growth rate, and its GRAS status (Claudia et al. 2017). In addition, *saccharomyces cerevisiae* has an inherent terpenoid production pathway, the mevalonate (MVA) pathway, in which isopentenyl diphosphate (IPP) or dimethylallyl diphosphate (DMAPP), essential intermediates of sterols and ubiquinones, are synthesized from acetyl-CoA (Guo et al. 2020). Metabolic reactions in the MVA pathway proceed in the cytosol, which, in turn, are used for terpenoid production. Recently, terpenoid production using organelles has attracted attention owing to their ability to produce acetyl-CoA.

Peroxisomes are considered potential acetyl-CoA pools because β-oxidation of fatty acids proceeds in them (Hammer and Avalos 2017). In fact, a terpenoid, β-amyrin, was produced at high level (57.8 mg/g dry cell weight (DCW)) by introducing two enzymes of the MVA pathway into peroxisomes and four downstream enzymes into the cytosol or peroxisomes (Du et al. 2022). The concentration of acetyl-CoA in the mitochondria is as much as 20–30 times higher than that in the cytosol because of the high enzymatic activity of the mitochondrial pyruvate dehydrogenase complex (Weinert et al. 2014). Thus, mitochondria are considered suitable organelles for the introduction of the MVA pathway for terpenoid production (Duran et al. 2020). For example, patchoulol, a terpenoid, was produced at a high level (19.24 mg/L) by introducing all the enzymes of the MVA pathway into the mitochondria and a fusion protein of the downstream enzymes into the cytosol (Tao et al. 2022). In another example, amorphadiene was produced at 400 mg/L by introducing several enzymes of the MVA pathway into the mitochondria and two downstream enzymes into the cytosol (Yuan and Ching 2016).

Although introducing the MVA pathway into the mitochondria is attractive for producing terpenoids in yeast, there may be a limit because the mitochondria comprise a small percentage of the entire cell (Uchida et al. 2011). We hypothesized that terpenoid production will increase by introducing the MVA pathway into the mitochondria when mitochondrial volume increases. In the present study, we constructed yeast strains to analyze squalene production or β-carotene production and investigated the relationship between mitochondria-mediated terpenoid (squalene or β-carotene) production and mitochondrial volume.

## Materials and methods

### Genes for yeast strain construction

The genes used for the construction of the yeast strains were prepared as described in the Supplementary Information. The plasmids, primers, and DNA fragments used are listed in Tables S1, S2, and S3, respectively. *Escherichia coli* NovaBlue (Merck Millipore, Darmstadt, Germany) was used for plasmid preparation using the Orthodox method.

### Yeast strains

*Saccharomyces cerevisiae* BY4741 was used as the host strain. The strains used in this study are listed in Table 1. The methods used for strain construction are described in the Supplementary Information.

**Table 1:** Strains used or constructed in this study

Strain	Genotype
BY4741	MATa, *his3*Δ*1*, *leu2*Δ*0*, *met15*Δ*0*, *ura3*Δ*0*
SSY1	BY4741, ARS208::T_TDH3_-*ERG10*-MLS-P_TDH3_-P_ADH1_-MLS-*ERG13*-T_ADH1_; ARS308::T_TDH3_-*tHMG1*-MLS-P_TDH3_-P_ADH1_-MLS-*ERG12*-T_ADH1_; ARS416::T_TDH3_-*ERG19*-MLS-P_TDH3_-P_ADH1_-MLS-*ERG8*-T_ADH1_
SSY2	SSY1, Δ*mdm32*
SSY3	SSY1, Δ*fzo1*
SSY4	SSY1, Δ*mgm1*
SSY5	SSY1, Δ*ugo1*
SSY6	SSY1, pCrtYBI-BTS1
SSY7	SSY1, Δ*mdm32*, pCrtYBI-BTS1
SSY8	SSY1, Δ*fzo1*, pCrtYBI-BTS1
SSY9	SSY1, Δ*mgm*, pCrtYBI-BTS1
SSY10	SSY1, Δ*ugo1*, pCrtYBI-BTS1

### Mitochondrial volume analysis

Mutants with defective mitochondrial morphology were selected from the Yeast Knockout Collection (Open Biosystems, Huntsville, AL, USA) database. The total mitochondrial volume per cell was quantified using a previously described method (Viana et al. 2015). The mutant strains were transformed with pGK426-MLS-GFP and cultured in 5 mL of synthetic complete media without uracil (2% glucose, 1.46 g/L Yeast Synthetic Drop-out Medium Supplements; Merck, Darmstadt, Germany) overnight at 30 °C with shaking at 200 rpm. The strains were transferred to YPD media (2% glucose, 2% peptone, and 1% yeast extract) at 0.1–0.4 OD_600_ and cultured further for 4 h under the same conditions. When the OD_600_ reached 0.5–1.0, the cultures were diluted 8–16-fold with YPD, transferred to a 96-well glass plate coated with concanavalin-A, and incubated at 30 °C for 20 min. The supernatant was removed and fresh YPD was added to each well. MLS-GFP signals in mutants were detected using an Olympus FV1000 confocal microscope (Olympus, Tokyo, Japan). Z-stack images of MLS-GFP were constructed and mitochondrial volumes were calculated using ImageJ and MitoGraph software. The mitochondrial volume in one cell was calculated as the average of at least 10 cells per strain.

### Analyses for IPP/DMAPP, squalene, and β-carotene contents

For squalene production, strains, pre-incubated in 5 mL of YPD at 30 °C overnight, were inoculated into 30 mL of YPD at 0.01 OD_600_ and incubated at 30 °C for 24 h in 250-mL baffled flask with shaking at 200 rpm. For β-carotene production, YPD supplemented with 200 μg/mL hygromycin (Nacalai Tesque, Japan) was used, and sampling was performed 48 h after inoculation. DCW was determined using the equation, DCW/OD_600_ = 0.561 derived beforehand.

Intracellular IPP/DMAPP were extracted as previously described (Luo et al. 2020). Briefly, 2 mL of each culture was centrifuged at 5,000 × *g* for 2 min, and the supernatant was removed. Cells were lysed in an acetonitrile/methanol/water (40:40:20) solution. The solution was chilled at –20 °C for 20 min, followed by centrifugation at 16,000 × *g* for 10 min. The supernatant was collected, dried, and resuspended in 50 μL of water. IPP/DMAPP were analyzed using liquid chromatography-tandem mass spectrometry (LC–MS/MS) (Agilent Nexera 1260 series high-performance liquid chromatography system and 6460 Triple Quad LC/MS; Agilent Technologies, Santa Clara, CA, USA). Separation, detection, and quantification of IPP /DMAPP were performed as previously described (Kato et al. 2012); however, IPP and DMAPP could not be separated from each other. Thus, in this study, the contents are presented as the sum of IPP and DMAPP (referred to as IPP/DMAPP).

Intracellular squalene was extracted as previously described (Zhu et al. 2021). Briefly, 600 μL of each culture was centrifuged at 5,000 × *g* for 2 min and the supernatant was removed. 600 μL of ethyl acetate and approximately 200 μL of grass beads (diameter of 0.5 mm) were added to the cells, which were then lysed by shaking at 1,500 rpm for 10 min using a grinder (shake Master NEO BMS, Japan) with a pre-cooled sample holder at 4 °C. The cell lysate was centrifuged at 21,880 × *g* for 10 min and the upper organic solvent was collected. A GCMS-QP2010 (Shimadzu, Japan) equipped with a DB-5MS column (15 m × 0.25 mm, 0.25 μm film thickness; Agilent Technologies) was used to analyze squalene. The oven temperature was maintained at 100 °C for 2 min, gradually increased to 250 °C at a rate of 20 °C/min, maintained for 2 min, then increased to 325 °C at a rate of 50 °C/min and maintained for 2 min.

Intracellular β-carotene was extracted as previously described (Ma et al. 2022). Briefly, 100 μL of each culture was centrifuged at 5,000 × *g* for 2 min and the supernatant was removed. A total of 900 μL of dimethyl sulfoxide was added to the cells, and β-carotene was extracted after incubation at 50 °C for 1 h. After the incubation, 450 μL of methanol was added and the mixtures were centrifuged at 21,880 × *g* for 5 min. The supernatants were collected. A high-performance liquid chromatography (HPLC) (Shimadzu, Japan) equipped with a BDS Hypersil C18 column (4.6 × 150 mm^2^, 5 μm particle size; Thermo Fisher Scientific, Waltham, MA, USA) and a SPD-20A UV–VIS detector (Shimadzu) was used to analyze β-carotene. The oven temperature was maintained at 25 °C. Methanol and acetonitrile were used as mobile phases at a flow rate of 0.28 mL/min and 0.52 mL/min. β-carotene was detected by absorbance at 450 nm.

The representative chromatograms of IPP/DMAPP, squalene, and β-carotene were shown in Figs, S1, S2, and S3.

### Measurement of intracellular oxidation levels

The reactive oxygen species (ROS) level was analyzed using ROS Assay Kit -Photo-oxidation Resistant DCFH-DA- (Dojindo, kumamoto, Japan). Briefly, strains, pre-incubated in 5 mL of YPD at 30 °C overnight, were inoculated into 5 mL of fresh YPD at 1.0 OD_600_ and incubated at 30 °C for 4 h in test tubes with shaking at 200 rpm. Then, 1 mL of each culture was centrifuged at 5,000 × *g* for 2 min, and the supernatant was removed. The cells were treated according to the manufacturer’s protocol and the fluorescence was measured with λ_ex_ = 500 nm and λ_em_ = 540 nm using a plate-reader (INFINITE M NANO + ; Tercan, Männedorf, Switzerland).

### Spot assay using dithiothreitol (DTT)

The effect of DTT addition to media on growth was analyzed as previously described (Bode et al. 2013). Briefly, strains, pre-incubated in 5 mL of YPD at 30 °C overnight, were inoculated at 1.0 OD_600_ into 5 mL of fresh YPD and incubated at 30 °C for 4 h in a test tube with shaking at 200 rpm. OD_600_ was adjusted to 1, and tenfold serial dilutions were spotted at 3μL onto YPD, YPD + DTT 0.5 mM, or YPD + DTT 5 mM plates. The plates were incubated at 30 °C for 24 h.

## Results

### The strain expressing the MVA pathway in mitochondria

**Fig. 1:**
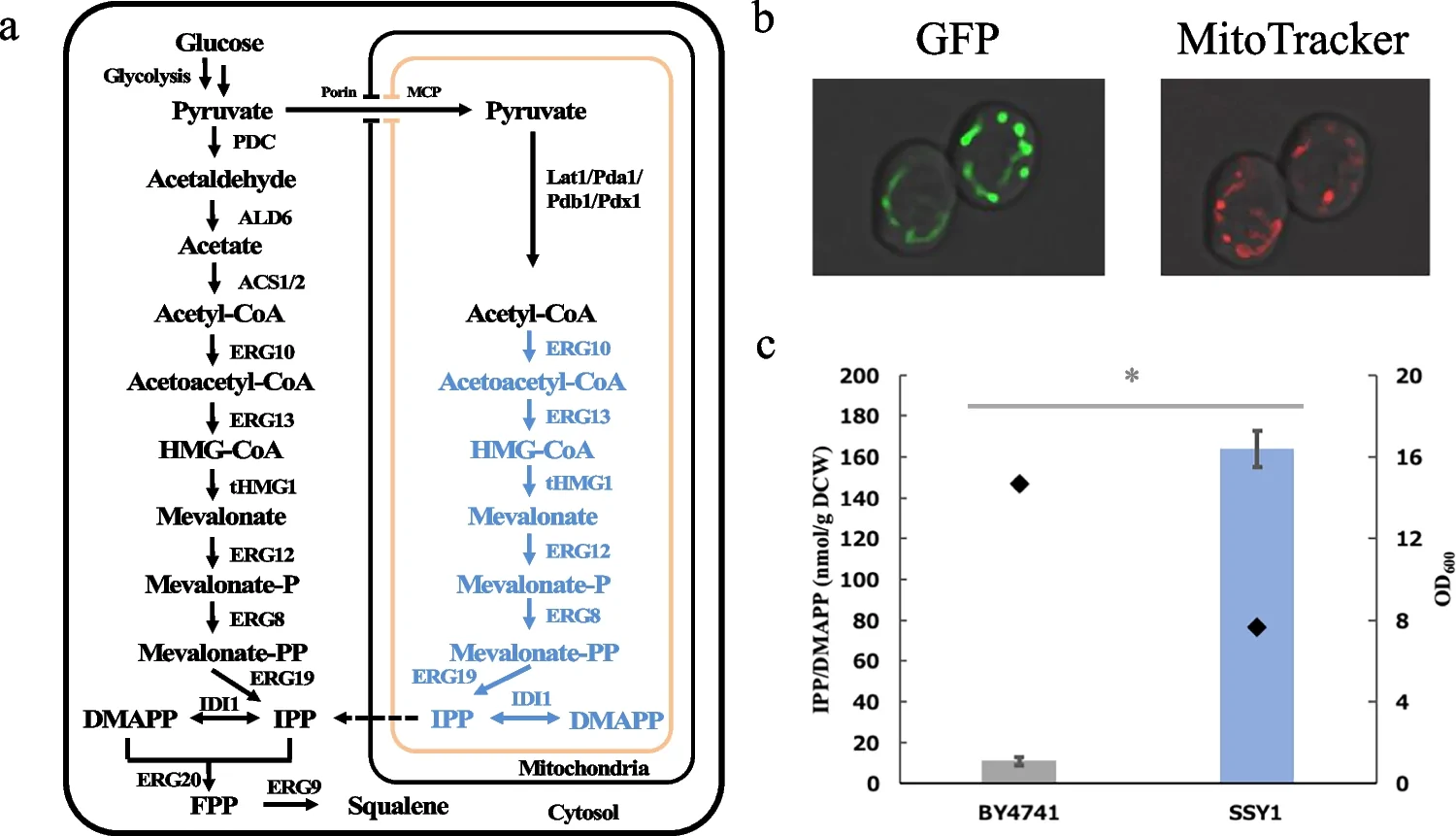
Introduction of the MVA pathway into mitochondria. **a** Introduction of the MVA pathway into mitochondria. Native and engineered pathways are shown in black and blue, respectively. **b** The localization of the MLS-fused GFP. The left and right panels show the fluorescent images of the MLS-fused GFP and MitoTracker. IPP production in BY4741 and SSY1. **c** The amount of IPP/DMAPP (columns) and OD_600_ (dots) were measured 24 h after inoculation. Data are presented as the mean ± standard deviation (SD). P-values were determined using two-tailed Student’s *t*-tests (* *P* < 0.05). Biological replication was achieved by three individual cultures

Proteins can be delivered to the mitochondria by fusion with a mitochondrial localization signal (MLS); however, their enzymatic activities are sometimes interrupted in the mitochondria if the MLS remains fused to them (Duran et al. 2020). To introduce the entire MVA pathway into the mitochondria and activate it, we constructed a BY4741 background strain, SSY1, expressing all the enzymes of the MVA pathway fused with the MLS of CoxIV (Fig. 1a), because it is cleaved away in mitochondria. The MLS of CoxIV consists of 26 amino acids; the first 25 amino acids and last glutamine are required for translocation and cleavage, respectively. To ascertain the action of MLS, we constructed a strain, expressing a GFP fused with MLS, and examined the localization of GFP as compared with MitoTracker. As shown in Fig. 1b, the signal of the MLS-fused GFP coincided with that of MitoTracker, confirming that the MLS of CoxIV worked effectively in translocation to the mitochondria. Thus, we hypothesized that all enzymes of the MVA pathway fused with MLS were also translocated to the mitochondria. Next, we examined whether the MVA pathway introduced into the mitochondria functioned correctly by measuring the amount of IPP/DMAPP in SSY1 compared to that in BY4741. The IPP/DMAPP content in SSY1 was 164 nmol/g DCW, which was 15-fold higher than that in BY4741, 11 nmol/g DCW (Fig. 1c). This result indicates that the MVA pathway introduced into the mitochondria functioned and was beneficial for terpenoid production, as previously reported (Lv et al. 2016).

### The total mitochondrial volumes in mutants with defective mitochondrial morphology

**Fig. 2:**
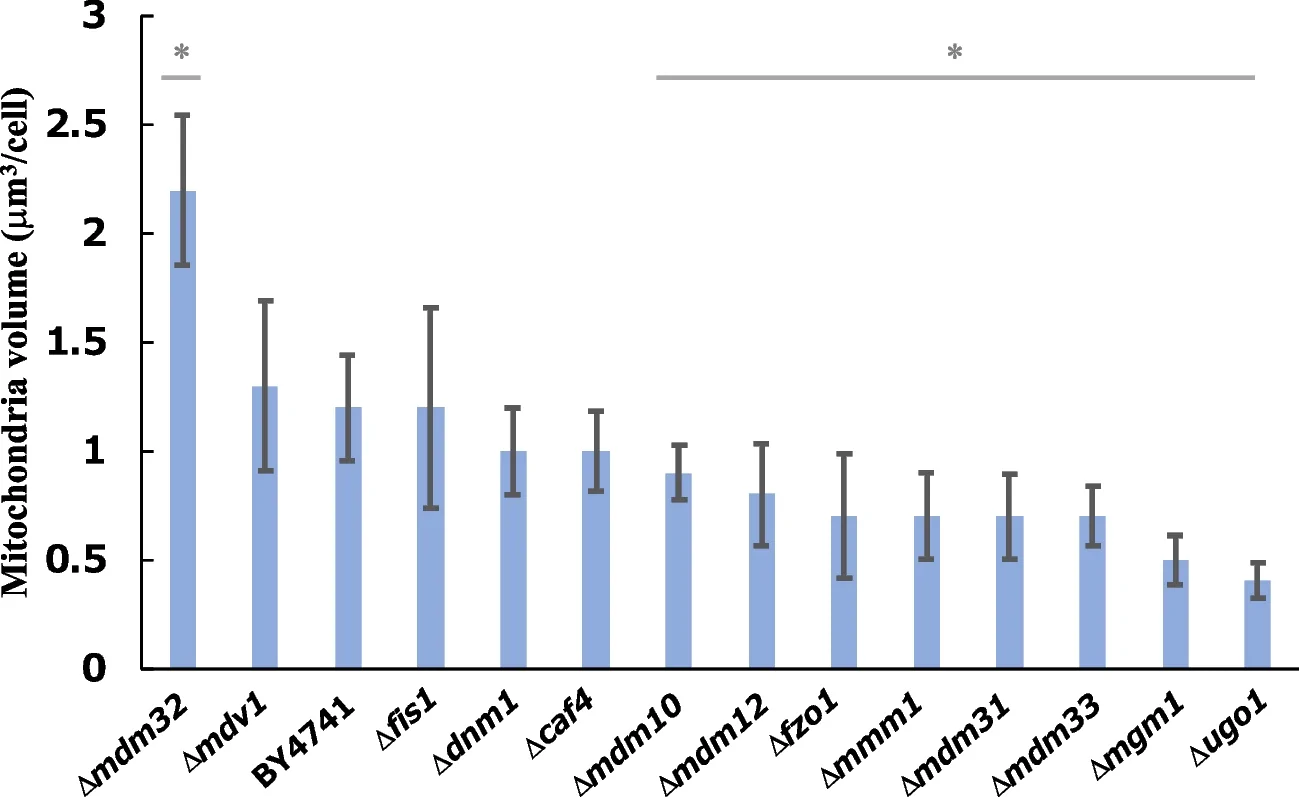
Mitochondrial volume in the mutants with defective mitochondrial morphology. All strains expressed MLS-fused GFP. Mitochondrial volume was calculated from the fluorescent signals of GFP using the confocal microscopic Z-stack method. Data are presented as the mean ± SD. *P*-values were determined using two-tailed Student’s *t*-tests (* *P* < 0.05) compared with BY4741 cells. Biological replication was achieved using at least ten individual cells

Mitochondrial volume has been reported to be decreased in a deletion mutant of *MGM1*, a mitochondrial morphology-related gene (Bernhardt et al. 2015). We hypothesized that mutants with defective mitochondrial morphology could be used to examine the effect of total mitochondrial volume on terpenoid production in yeast expressing the MVA pathway in their mitochondria. We selected 13 genes, *FZO1*, *MGM1*, *UGO1*, *DNM1*, *FIS1*, *MDV1*, *CAF4*, *MMM1*, *MDM10*, *MDM12*, *MDM31*, *MDM32*, and *MDM33* (Ohsumi and Shimoda 2007). GFP fused with the MLS of CoxIV was expressed in BY4741 and the deletion mutants, and mitochondrial volumes were calculated from the fluorescent signals of GFP using the confocal microscopic Z-stack method (Fig. 2). As expected, we found differences in mitochondrial volume among them. The only strain with a higher volume than that in BY4741 was Δ*mdm32* (2.2 m^3^/cell), which was 1.8-fold higher than that in BY4741 (1.2 μm^3^/cell). The strains with volumes lower than those in BY4741 were Δ*mdm10*, Δ*mdm12*, Δ*fzo1*, Δ*mmm1,* Δ*mdm31,* Δ*mdm33,* Δ*mgm1*, and Δ*ugo1*. The strain with the lowest volume was Δ*ugo1* (0.4 μm^3^/cell), which was threefold lower than that in BY4741.

### The effect of mitochondrial volume on mitochondria-mediated terpenoid production

**Fig. 3:**
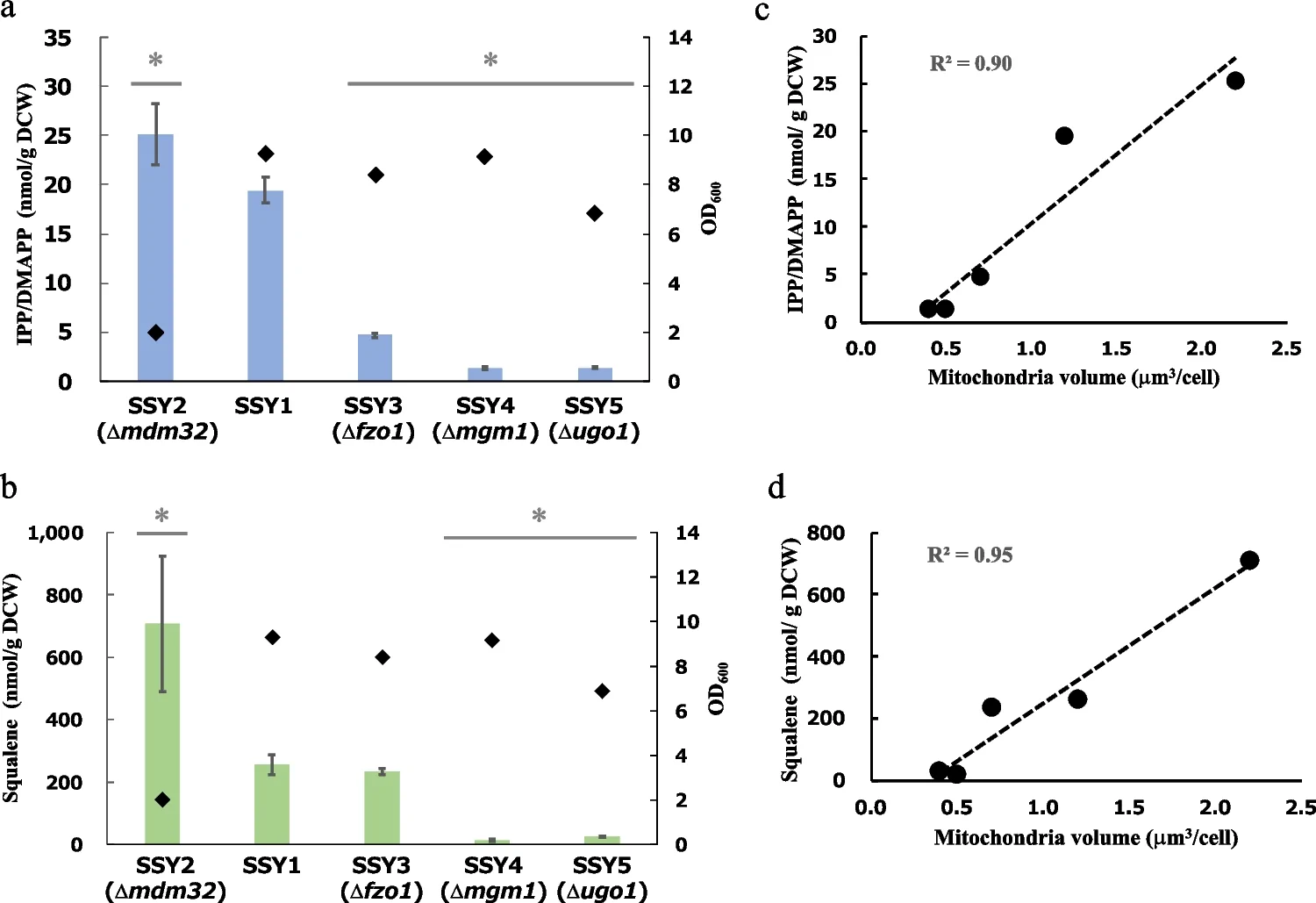
Effect of mitochondrial volume on squalene production. **a** Effect of mitochondrial volume on IPP/DMAPP level. The amounts of IPP/DMAPP (columns) and OD_600_ (dots) were measured 24 h after inoculation. Biological replication was achieved using three individual cultures. *P*-values were determined using two-tailed Student’s *t*-tests (* *P* < 0.05) compared with SSY1. **b** Squalene production; the amount of squalene (columns) and OD_600_ (dots) were measured 24 h after inoculation. Biological replication and P-values were the same in (**a**). **c** The relationship between the amount of IPP/DMAPP and mitochondrial volume. **d** The relationship between squalene production and mitochondrial volume

We hypothesized that an increase in mitochondrial volume may augment the terpenoid production in yeast expressing the MVA pathway in their mitochondria. To examine the effect of mitochondrial volume on terpenoid production, we used mutants with defective mitochondrial morphology, Δ*mdm32*, Δ*fzo1*, Δ*mgm1*, and Δ*ugo1*. Four mutants were constructed from SSY1, and the relationship between mitochondrial volume and IPP/DMAPP or squalene content was examined. In SSY2 (Δ*mdm32*) harboring a large mitochondrial volume, both the contents of IPP/DMAPP (25 nmol/g DCW) and squalene (707 nmol/ g DCW) increased 1.3- and 2.8-fold higher than that in SSY1 (19 nmol/g DCW for IPP/DMAPP, 256 nmol/ g DCW for squalene) (Fig. 3a and b). In contrast, in SSY3 (Δ*fzo1*), SSY4 (Δ*mgm1*), and SSY5 (Δ*ugo1*) harboring a low of mitochondrial volume, the contents of IPP/DMAPP and squalene decreased compared with that in SSY1, with the exception of squalene in SSY3. The contents of IPP/DMAPP in SSY3, 4, and 5 were 4.65, 1.29, and 1.32 nmol/g DCW, which were 4.2-, 15.1-, and 14.7-fold less than those in SSY1. The concentrations of squalene in SSY3, 4, and 5 were 233, 14, and 25 nmol/ g DCW, which were 1.1-, 18.2-, and 10.1-fold less than those in SSY1. Furthermore, we found that the contents of both IPP/DMAPP and squalene were clearly correlated with the mitochondrial volume (Fig. 3c and d).

**Fig. 4:**
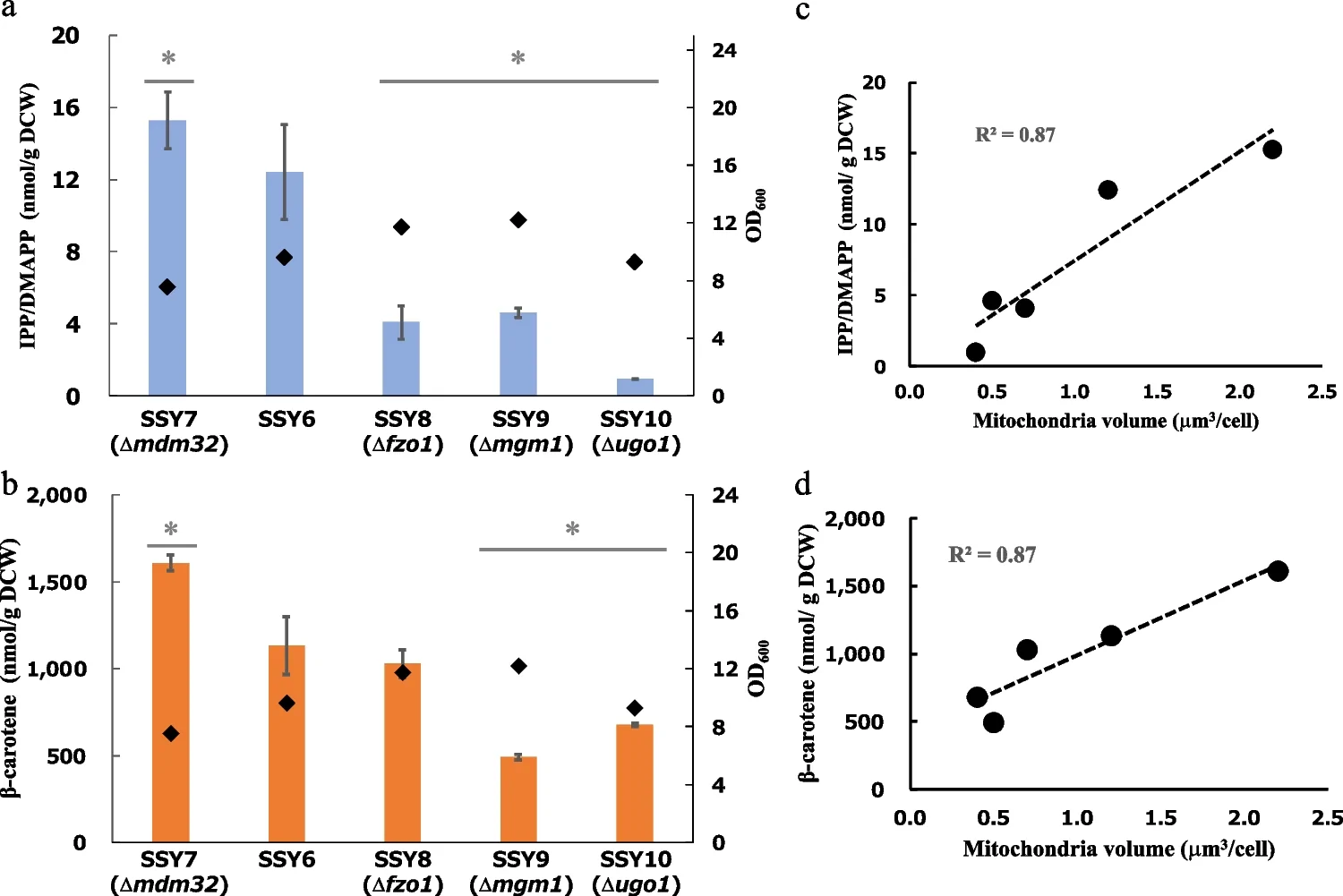
Effect of mitochondrial volume on β-carotene production. **a** Effect of mitochondrial volume on IPP/DMAPP level. The amounts of IPP/DMAPP (columns) and OD_600_ (dots) were measured 48 h after inoculation. Biological replication was achieved using three individual cultures. P-values were determined using two-tailed Student’s *t*-tests (* P < 0.05) compared with SSY6. **b** β-carotene production; the amount of β-carotene (columns) and OD_600_ (dots) were measured 48 h after inoculation. Biological replication and P-values were the same in (**a**). **c** The relationship between the amount of IPP/DMAPP and mitochondrial volume. **d** The relationship between β-carotene production and mitochondrial volume

In addition, we investigated the effect of the mitochondrial volume on β-carotene production to evaluate the effectiveness of our approach on other terpenoid production. In SSY7 (Δ*mdm32*) harboring a large mitochondrial volume, the contents of IPP/DMAPP (15 nmol/g DCW) and β-carotene (1609 nmol/ g DCW) were 1.2- and 1.4-fold higher than those in SSY6 (12.4 nmol/g DCW for IPP/DMAPP, 1132 nmol/ g DCW for β-carotene) (Fig. 4a and b). In contrast, the contents of IPP/DMAPP in SSY8 (Δ*fzo1*), 9 (Δ*mgm1*), and 10 (Δ*ugo1*) were 3.0-, 2.7-, and 12.4-fold lower than those in SSY6 at 4.1, 4.6, and 1.0 nmol/g DCW, respectively. The concentrations of β-carotene in SSY8, 9, and 10 were 1030, 491, and 678 nmol/ g DCW, 1.1-, 2.3-, and 1.7-fold lower than those in SSY6, respectively. Furthermore, the contents of both IPP/DMAPP and β-carotene were correlated with the mitochondrial volume (Fig. 4c and d). These data indicate that the increase in mitochondrial volume augmented terpenoid production in yeast expressing the MVA pathway in the mitochondria.

## Discussion

In this study, we demonstrated the beneficial effect of the MVA pathway introduced into the mitochondria for terpenoid production by measuring IPP/DMAPP, an essential precursor of terpenoids. Furthermore, using mutants with defective mitochondrial morphology, we found that the contents of IPP/DMAPP and terpenoids (squalene or β-carotene) were positively correlated with the mitochondrial volume and showed differences in the mitochondrial volume. These results not only support the advantages of introducing the MVA pathway into mitochondria for terpenoid production but also suggest that increasing the mitochondrial volume might enhance the production.

Both IPP/DMAPP and terpenoids contents were positively correlated with the mitochondrial volume. However, notably, there was a difference in the degree of increase between IPP/DMAPP and terpenoids in SSY2 (Δ*mdm32*) and SSY7 (Δ*mdm32*) (1.3-fold for IPP/DMAPP and 2.8-fold for squalene in squalene production, 1.2-fold for IPP/DMAPP and 1.4-fold for in β-carotene production). IDI1 was not introduced into the mitochondria of any of the strains used in this study. Thus, DMAPP was not synthesized in the mitochondria. The growth defect compared to the wild-type strain is believed to be caused by the accumulation of the pyrophosphate compounds, mevalonate-5-PP, IPP, and DMAPP in the mitochondria (Zhu et al. 2021), which was also observed in this study (Fig. 1 C). Pyrophosphate compounds can react with adenosine monophosphate-amino acids through aminoacyl tRNA synthetases and convert them to toxic ATP analogs, which inhibit mitochondrial adenine nucleotide translocase and F1-ATPase (Mönkkönen et al. 2006, Mookerjee-Basu et al. 2010). However, we first speculated that the defective mitochondrial morphology or the introduction of MVA pathway enzymes into the mitochondria might cause mitochondrial dysfunction and induce oxidative stress. Therefore, we measured the ROS levels and growth of BY4741, *mdm32* deletion strain, SSY1, and SSY2 (Δ*mdm32*) and found that the ROS levels in SSY2 (Δ*mdm32*) tended to be slightly higher compared to those in BY4741 (Fig. S4). Moreover, the addition of DTT as a reducing agent to media improved the growth of the strain, which was exposed to oxidative stress (Bode et al. 2013). Thus, a DTT assay was performed using the SSY2 (Δ*mdm32*) strain, which revealed that DDT addition to YPD media did not improve the growth of SSY2 (Δ*mdm32*) (Fig. S5). Therefore, the slight increase in ROS levels in SSY2 (Δ*mdm32*) was not the cause of the growth defect of SSY2 (Δ*mdm32*), supporting the theory that growth defects are attributed to the accumulation of the pyrophosphate compounds mentioned in the previous study (Zhu et al. 2021).

Thus, it is plausible that IPP/DMAPP mainly contains IPP accumulated in the mitochondria. After production in the mitochondrial matrix, IPP is exported to the cytosol, where squalene production proceeds (Zhu et al. 2021). During export, IPP must pass through the inner and outer mitochondrial membranes. Several transporters selectively pass through small molecules in the inner membranes (Duran et al 2020). Porin is present in the outer membrane and allows small molecules (< 5 kDa) to pass freely between the cytosol and intermembrane space. Thus, passage through the inner membrane is thought to be critical for IPP export from the mitochondria to the cytosol. Mdm32 is a protein present in the mitochondrial inner membrane, and loss of Mdm32 leads to defects in mitochondrial morphology (Okamoto and Shaw 2005). Although the mechanism underlying the IPP export remains to be elucidated, structural changes in the inner mitochondrial membrane may enhance IPP export or diffusion.

Regarding the Δ*fzo1* strains (SSY3 (Δ*fzo1*) and SSY8 (Δ*fzo1*)), although both IPP and terpenoid decreased in the Δ*fzo1* strains compared to the normal strains (SSY1 and SSY6), we also observed a difference in the degree of decrease between IPP and squalene content: 76% for IPP and 9% for squalene (Fig. 3a and c) and that between IPP and β-carotene content: 68% for IPP and 9% for β-carotene (Fig. 4a and c). These differences seem to arise from the balance between IPP synthesis in the mitochondria and IPP export. Due to the low mitochondrial volume, a sufficient amount of enzymes in the MVA pathway could not be transported to the mitochondria; thus, the rate of IPP synthesis decreased. The IPP produced via the mitochondria in the Δ*fzo1* strains seem to be exported smoothly to the cytosol, because of its low synthetic activity, and is subsequently metabolized to squalene or β-carotene. Thus, although the terpenoid level in the Δ*fzo1* strains was slightly lower than that in the normal strains, the difference in terpenoid levels between the normal strains and the Δ*fzo1* strains was not as drastic as that in IPP. In contrast, the IPP/DMAPP levels in the Δ*fzo1* strains decreased drastically compared with those in the normal strains (Figs. 3a and 4 a). The Fzo1 protein resides in the outer mitochondrial membrane, and the loss of *Fzo1* may not affect IPP export or diffusion due to the presence of porins in the outer membrane, suggesting that there may be no difference in the export ability of mitochondria. There may be a limit to export, and the IPP in the normal strains probably accumulates in the mitochondrial matrix.

In the present study, SSY1 to 10 showed growth defects compared to the wild-type strain, BY4741. The defects were severe in the Δ*mdm32* strains (SSY2 and SSY7). The deletion of *FZO1*, *MGM1*, *UGO1*, or *MDM32* leads growth defects by dysfunction of mitochondria (Okamoto and Shaw 2005). In the normal and Δ*mdm32* strains, the IPP/DMAPP levels were high (Figs. 3a and 4a), indicating that pyrophosphate compounds may accumulate in the mitochondria. Therefore, the severe growth defects in the Δ*mdm32* strains is probably due to both mitochondrial dysfunction and pyrophosphate compound accumulation in the mitochondria. As the normal strains had no mitochondrial morphological defects. Thus, the growth defect in the normal strains may only be due to the accumulation of pyrophosphate compounds in the mitochondria. In contrast, the growth defects in Δ*fzo1* strains, Δ*mgm1* strains (SSY4 and SSY9), and Δ*ugo1* strains (SSY5 and SSY10) may be due to mitochondrial dysfunction because their IPP levels, that is, their activities in the MVA pathway introduced in the mitochondria, were considerably low. Further approaches to expand the volume of mitochondria without growth defects by mitochondrial dysfunction and enhance the export of IPP, inhibiting pyrophosphate compound accumulation, would be required to increase terpenoid production using our proposed mitochondria-based strategy.

## Supplementary Information

Below is the link to the electronic supplementary material.

Supplementary file1 (PDF 1399 KB)

## Data Availability

The data generated and/or analyzed during the current study are available from the corresponding author upon reasonable request.
