# Supplementary material for: The potency of mitochondria enlargement for mitochondria-mediated terpenoid production in yeast
Source: Appl Microbiol Biotechnol. 2024 Jan 13;108:110. doi: 10.1007/s00253-023-12922-5 (PMC10787878; doi:10.1007/s00253-023-12922-5)
Supplement: Supplementary file 1 [file 253_2023_12922_MOESM1_ESM.pdf]

Applied Microbiology and Biotechnology

The potency of mitochondria enlargement for mitochondria-mediated terpenoid production in yeast

So Yanagibashi[1,2] , Takahiro Bamba[3] , Takayoshi Kirisako[2] , Akihiko Kondo[1,3,4] , Tomohisa Hasunuma[1,3,4*]

[1] Graduate School of Science, Technology and Innovation, Kobe University, 1-1 Rokkodai, Nada, Kobe 657-8501, Japan

[2] Kirin Central Research Institute, Kirin Holdings Company, Ltd., 26-1-12-12 Muraoka-Higashi 2-chome, Fujisawa, Kanagawa 251-8555, Japan

[3] Engineering Biology Research Center, Kobe University, 1-1 Rokkodai, Nada, Kobe 6578501, Japan

[4] Biomass Engineering Program, RIKEN, 1-7-22 Suehiro-cho, Tsurumi-ku, Yokohama, Kanagawa 230-0045, Japan

*Corresponding Author:

Tomohisa Hasunuma, Engineering Biology Research Center, Kobe University, 1-1 Rokkodai, Nada, Kobe 657-8501, Japan; orcid.org/0000-0002-8382-2362

Phone/Fax: +81-78-803-6461

Email: hasunuma@port.kobe-u.ac.jp

Plasmid construction

The plasmids, the primers and the DNA fragments used in this study are listed in Table S1, S2 and S3 respectively. Unless otherwise noted, all of the plasmids were constructed by using In-fusion HD cloning Kit (Takara Bio USA, Mountain View, CA, USA), according to the manufacturer's protocol.

1. pGK426-MLS-GFP, the plasmid expressing the MLS-GFP

A DNA fragment 1 of partial MLS-fused GFP was amplified using primer 1 and 3 and a template, pGK416-ymUkG1 (Kaishima 2016). Using primer 2 and 3 and the DNA fragment 1 amplified as the template, the DNA fragment 2 of the MLS-GFP was amplified and cloned into pGK426 at the site between SalI and BamHI.

2. pPreARS208, the plasmid to prepare for construction of pARS208-*ERG13-ERG10*

The ARS208 upstream region, promoter and terminator set (TDH3 promoter, TDH3 terminator, ADH1 promoter and ADH1 terminator) and ARS208 downstream region were amplified using *S.cereviciae* BY4741 genome and pATP426 (Ishii 2014) as the template and primer 4 to 9. The DNA fragments 3 to 5 were cloned to pUC19 digested by HindIII and NdeI. The resulting plasmid was designated as pPreARS208.

3. pARS208-*ERG13-ERG10*, the plasmid to prepare for construction of pARS208-*ERG13-ERG10-URA3*

A DNA fragment 6 of partial MLS-fused *ERG13* was amplified using primer 20 and 21 and BY4741 genome as a template. Using primer 20 and 22 and the DNA fragment 7 amplified as the template, the DNA fragment of the MLS-*ERG13* was amplified. Similarly, a DNA fragment 8 of partial MLS-fused *ERG10* was amplified using primer 25 and 27 and BY4741 genome, and, using primer 26 and 27 and the DNA fragment 8 amplified as the template, the DNA fragment 9 of the MLS- *ERG10* was amplified.

The DNA fragment 10 containing TDH3 promoter for the MLS-*ERG13* and ADH1 promoter for the MLS-*ERG10* was amplified using primer 23 and 24 and pATP426 as the template. The DNA fragments 7,9 and 10 were cloned to pPre208 digested by PmeI and MluI. The resulting plasmid was designated as pARS208-*ERG13-ERG10*.

4. pARS208-*ERG13-ERG10-URA3*, the plasmid for integration of the genes encoding MLS-*ERG13* and MLS-*ERG10* into genome

The DNA fragment 11 of the *URA3* gene was amplified using primer 36 and 37 and pGK426 as the template. The DNA fragment 11 was cloned to pARS208-*ERG13-ERG10* digested by SacII. The resulting plasmid was designated as pARS208-*ERG13-ERG10-URA3*

5. pPreARS308, the plasmid to prepare for construction of pARS308-*ERG12-tHMG1*

The ARS308 upstream region, promoter and terminator set (TDH3 promoter, TDH3 terminator, ADH1 promoter and ADH1 terminator) and ARS308 downstream region were amplified using *S.cereviciae* BY4741 genome and pATP426 as the template and primer 7 and 11 to 14. The DNA fragments 12 to 14 were cloned to pUC19 digested by HindIII and NdeI. The resulting plasmid was designated as pPreARS308.

6. pARS308-*ERG12-tHMG1*, the plasmid to prepare for construction of pARS308-*ERG12-tHMG1-URA3*

A DNA fragment 15 of partial MLS-fused *ERG12* was amplified using primer 28 and 29 and BY4741 genome as a template. Using primer 22 and 28 and the DNA fragment 15 amplified as the template, the DNA fragment 16 of the MLS-*ERG12* was amplified. Similarly, a DNA fragment 17 of partial MLS-fused *tHMG1* was amplified using primer 30 and 31 and BY4741 genome, and, using primer 26 and 31 and the DNA fragment 17 amplified as the template, the DNA fragment 18 of the MLS- *tHMG1* was amplified.
The DNA fragment 10 containing TDH3 promoter for the MLS-*ERG12* and ADH1 promoter for the MLS-*tHMG1* was amplified using primer 23 and 24 and pATP426 as the template. The DNA fragments 10,16 and 18 were cloned to pPre308 digested by PmeI and MluI. The resulting plasmid was designated as pARS308-*ERG12-tHMG1*.

7. pARS308-*ERG12-tHMG1-URA3*, the plasmid for integration of the genes encoding MLS-*ERG12* and MLS-*tHMG1* into genome

The DNA fragment 11 of the *URA3* gene was amplified using primer 36 and 37 and pGK426 as the template. The DNA fragment 11 was cloned to pARS308-*ERG12-tHMG1* digested by SacII. The resulting plasmid was designated as pARS308-*ERG12-tHMG1-URA3*

8. pPreARS416, the plasmid to prepare for construction of pARS416-*ERG19-ERG8*

The ARS416 upstream region, promoter and terminator set (TDH3 promoter, TDH3 terminator, ADH1 promoter and ADH1 terminator) and ARS416 downstream region were amplified using *S.cereviciae* BY4741 genome and pATP426 as the template and primer 7 and 15 to 19. The DNA fragments 19 to 21 were cloned to pUC19 digested by HindIII and NdeI. The resulting plasmid was designated as pPreARS416.

9. pARS416-*ERG19-ERG8*, the plasmid to prepare for construction of pARS416-*ERG19-ERG8-URA3*

A DNA fragment 22 of partial MLS-fused *ERG19* was amplified using primer 32 and 33 and BY4741 genome as a template. Using primer 22 and 32 and the DNA fragment 22 amplified as the template, the DNA fragment 23 of the MLS-*ERG19* was amplified. Similarly, a DNA fragment 24 of partial MLS-fused *ERG8* was amplified using primer 34 and 35 and BY4741 genome, and, using primer 26 and 35 and the DNA fragment 24 amplified as the template, the DNA fragment 25 of the MLS-*ERG8* was amplified.

The DNA fragment 26 containing TDH3 promoter for the MLS-*ERG19* and ADH1 promoter for the MLS-*ERG8* was amplified using primer 23 and 24 and pATP426 as the template. The DNA fragments 10, 23 and were cloned to pPre308 digested by PmeI and MluI. The resulting plasmid was designated as pARS416-*ERG19-ERG8*.

10. pARS416-*ERG19-ERG-URA3*, the plasmid for integration of the genes encoding MLS-*ERG19* and MLS-*ERG8* into genome

The DNA fragment 11 of the *URA3* gene was amplified using primer 36 and 37 and pGK426 as the template. The DNA fragment 11 was cloned to pARS416-*ERG19-ERG8* digested by SacII. The resulting plasmid was designated as pARS416-*ERG19-ERG8-URA3*

11. p*Fzo1*, the plasmid to prepare for construction of p*Fzo1-URA3* and remove *URA3* integrated into genome

The *Fzo1* upstream region and downstream region were amplified using *S.cereviciae* BY4741 genome and primer 40 to 43. The DNA fragments 26 and 27 were cloned to pUC19 digested by digested by HindIII and NdeI. The resulting plasmid was designated as p*Fzo1*.

12. p*Fzo1-URA3*, the plasmid for deletion of *Fzo1*

The *Fzo1* upstream region, *URA3* gene and *Fzo1*downstream region were amplified using *S.cereviciae* BY4741 genome and primer 40, 43 56 to 59. The DNA fragments 28 to 30 were cloned to pUC19 digested by digested by HindIII and NdeI. The resulting plasmid was designated as p*Fzo1-URA3*.

13. p*Mgm1*, the plasmid to prepare for construction of p*Mgm1-URA3* and remove *URA3* integrated into genome

The *Mgm1* upstream region and downstream region were amplified using *S.cereviciae* BY4741 genome and primer 44 to 47. The DNA fragments 31 and 32 were cloned to pUC19 digested by digested by HindIII and NdeI. The resulting plasmid was designated as p*Mgm1*.

14. p*Mgm1-URA3*, the plasmid for deletion of *Mgm1*

The *Mgm1* upstream region, *URA3* gene and *Mgm1* downstream region were amplified using *S.cereviciae* BY4741 genome and primer 40, 43 57, 58, 60 and 61. The DNA fragments 29, 33 and 34 were cloned to pUC19 digested by digested by HindIII and NdeI. The resulting plasmid was designated as p*Mgm1-URA3*.

15. p*Ugo1*, the plasmid to prepare for construction of p*Ugo1-URA3* and remove *URA3* integrated into genome

The *Ugo1* upstream region and *Ugo1* downstream region were amplified using *S.cereviciae* BY4741

genome and primer 48 to 51. The DNA fragments 35 and 36 were cloned to pUC19 digested by digested by HindIII and NdeI. The resulting plasmid was designated as p*Ugo1*.

16. p*Ugo1-URA3*, the plasmid for deletion of *Ugo1*

The *Ugo1* upstream region, *URA3* gene and *Ugo1* downstream region were amplified using *S.cereviciae* BY4741 genome and primer 40, 43 57, 58, 62 and 63. The DNA fragments 29,37 and 38 were cloned to pUC19 digested by digested by HindIII and NdeI. The resulting plasmid was designated as p*Ugo1-URA3*.

17. p*Mdm32*, the plasmid to prepare for construction of p*Mdm32-URA3* and remove *URA3* integrated into genome

The *Mdm32* upstream region and downstream region were amplified using *S.cereviciae* BY4741 genome and primer 52 to 55. The DNA fragmnts 39 and 40 were cloned to pUC19 digested by digested by HindIII and NdeI. The resulting plasmid was designated as p*Mdm321*.

18. p*Mdm32-URA3*, the plasmid for deletion of *Mdm32*

The *Mdm32* upstream region, *URA3* gene and *Mdm32* downstream region were amplified using *S.cereviciae* BY4741 genome and primer 52, 55, 57, 58, 64 and 65. The DNA fragments 29, 41 and 42 were cloned to pUC19 digested by HindIII and NdeI. The resulting plasmid was designated as p*Mdm32-URA3*.

19. pCrtYBI-BTS1, the plasmid for expressing CrtYBI and overexpressing BTS1

The hygromycin B resistance gene region was amplified using pRDH227 plasmid and primer 74 and 75. The DNA fragment was cloned to pATP416-crtYBI digested by NsiI and SbfI. The resulting plasmid was designated as pCrtYBI-BTS1.

Construction of recombinant yeast strain

Yeast strains used in this study are listed in Table 1. *S.cereviciae* transformation was carried out by using a lithium acetate transformation method, as previously reported (Chen 1992) . The resulting transformants were spread on SD-URA or SD with 5-fluoroorotic acid (5-FOA) agar plate.

1. Construction of SSY1

pARS208-*ERG13*-*ERG10*-*URA3* was linearized with PacI, transfected into the BY4741, and integrated in the ARS208 region by double crossover recombination, yielding BY4741, ARS208::T_{TDH3}-*ERG10*-MLS-P_{TDH3}-*URA3*-P_{ADH1}-MLS-*ERG13*-T_{ADH1} strain. Then, a DNA fragment 43 composed of TDH3 promoter and ADH1 promoter was amplified using the pATP426 as the template and primer 38 and 39. To remove the *URA3* gene from BY4741, ARS208::T_{TDH3}-*ERG10*-MLS-P_{TDH3}-*URA3*-P_{ADH1}-MLS-*ERG13*-T_{ADH1} strain, the donor DNA was transfected into the BY4741, ARS208::T_{TDH3}-*ERG10*-MLS-P_{TDH3}-*URA3*-P_{ADH1}-

MLS-*ERG13*-T_{ADH1} strain and BY4741, ARS208::T_{TDH3}-*ERG10*-MLS-P_{TDH3}-P_{ADH1}-MLS-*ERG13*-T_{ADH1} strain was obtained. The same method was applied sequentially using pARS308-*ERG12-tHMG1-URA3* and pARS416-*ERG19-ERG8-URA3* to construct SSY1.

2. Construction of SSY2 to SSY5

A DNA fragment 44 composed of the *Fzo1* upstream region, *URA3* gene and *Fzo1* downstream region was amplified using p*Fzo1-URA3* as the template and primer 66 and 67, transfected into the SSY1, yielding SSY1 Δ*Fzo1*::*URA3*. Then, a DNA fragment 45 of *Fzo1* upstream region and downstream region was amplified using p*Fzo1* as the template and primer 66 and 67. To remove the *URA3* gene from SSY1 Δ*Fzo1*::*URA3*, the DNA fragment 45 was transfected into the SSY1 Δ*Fzo1*::*URA3* and SSY2 was obtained. The same method was applied using the DNA fragments 46 and 47 or 48 and 49 or 50 and 51 to construct SSY3 to SSY5.

3. Consturuction of SSY6 to SSY10

pCrtYBI-BTS1 was transfected into the SSY1 to SSY5, yielding SSY6 to SSY10.

Reference

1. Chen DC, Yang BC, Kuo TT, (1992) One-step transformation of yeast in stationary phase. *Curr Genet*, 21, 83–84. https://doi.org/10.1007/BF00318659.
2. Xiaochen H, Shunwen B, Zhuo L, Tomohisa H, Akihiko K, Shin-Hsin H (2020) Fermentation of pigment-extracted microalgal residue using yeast cell-surface display: Direct high-density ethanol production with competitive life cycle impacts. *Green Chem*, 22:153-162, https://doi.org/10.1039/C9GC02634G
3. Jun I, Takashi K, Harumi M, Akira O, Fumio M, Akihiko K (2014) Three gene expression vector sets for concurrently expressing multiple genes in *Saccharomyces cerevisiae. FEMS Yeast Research*, 14:399–411, https://doi.org/10.1111/1567-1364.12138.
4. Misato K, Jun I, Toshihide M, Nobuo F, Akihiko K (2016) Expression of varied GFPs in Saccharomyces cerevisiae: codon optimization yields stronger than expected expression and fluorescence intensity. *Sci Rep*, 6:35932, https://doi.org/10.1038/srep35932.
5. Masahiro T, Kenta N, Daisuke U, Jun I, Akihiko K (2021) Robust and flexible platform for directed evolution of yeast genetic switches. *Nat Commun*, 12:1846, https://doi.org/10.1038/s41467-021-22134-y

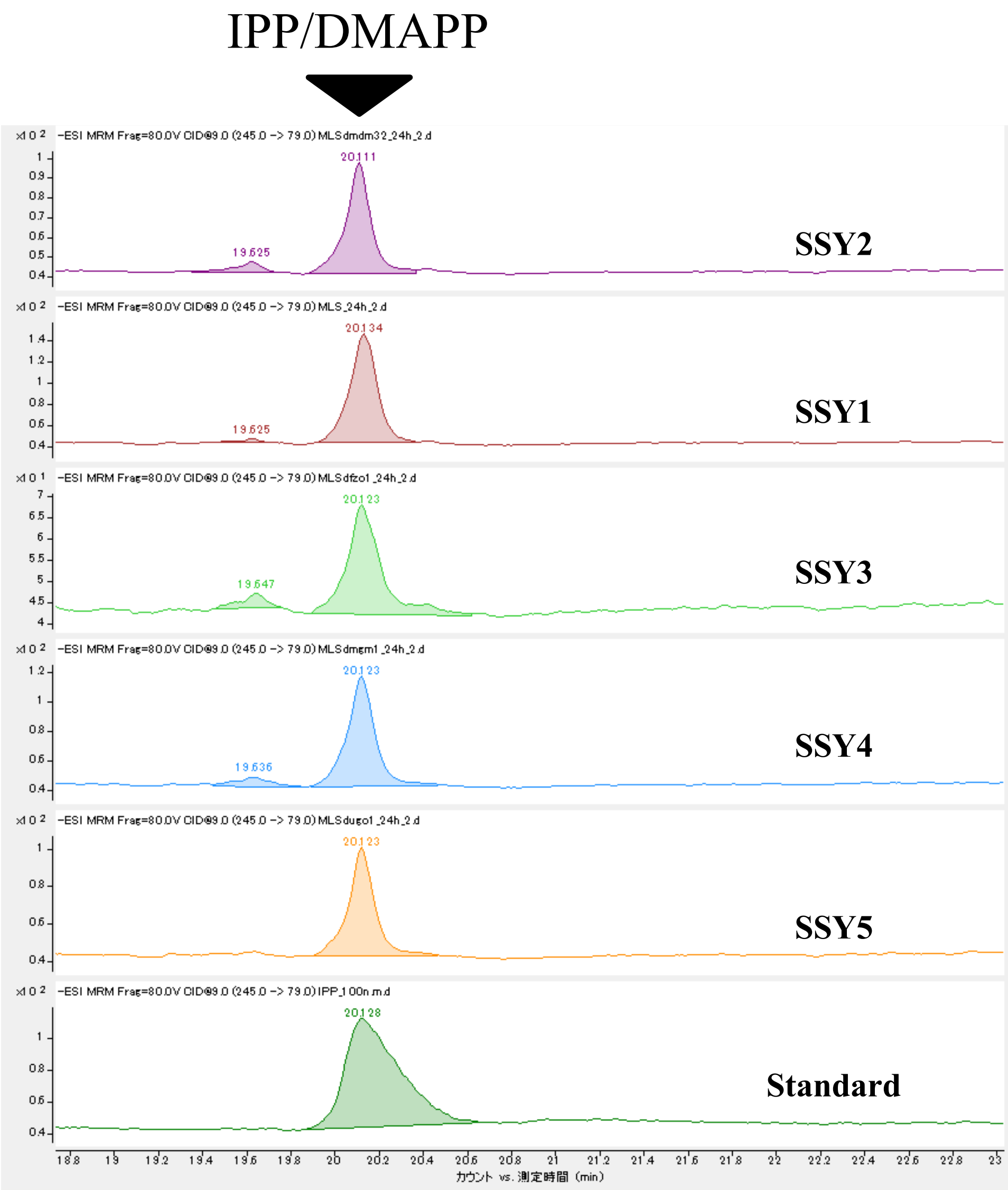

Fig. S1 The representative chromatograms of IPP/DMAPP

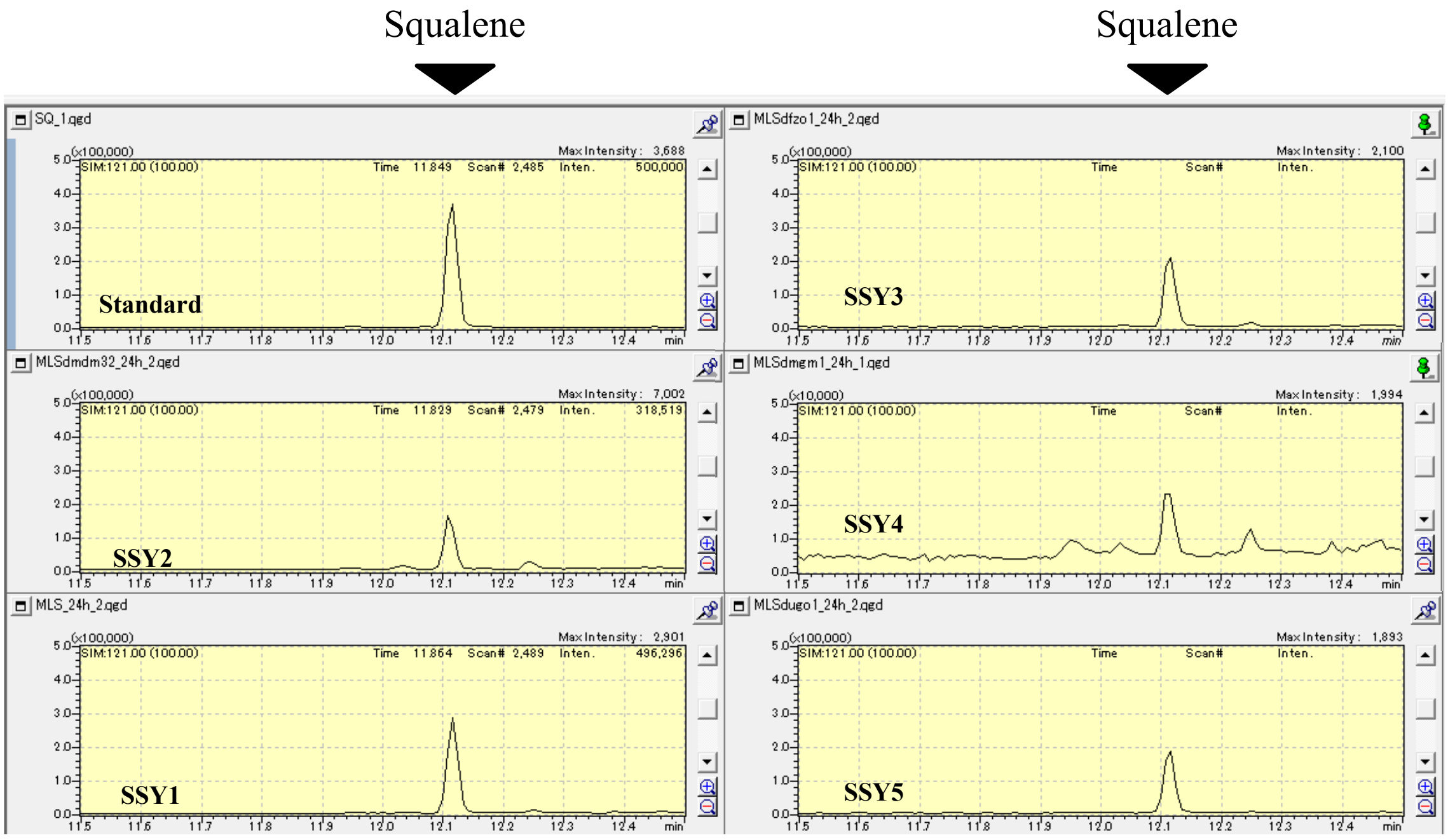

Fig. S2 The representative chromatograms of squalene

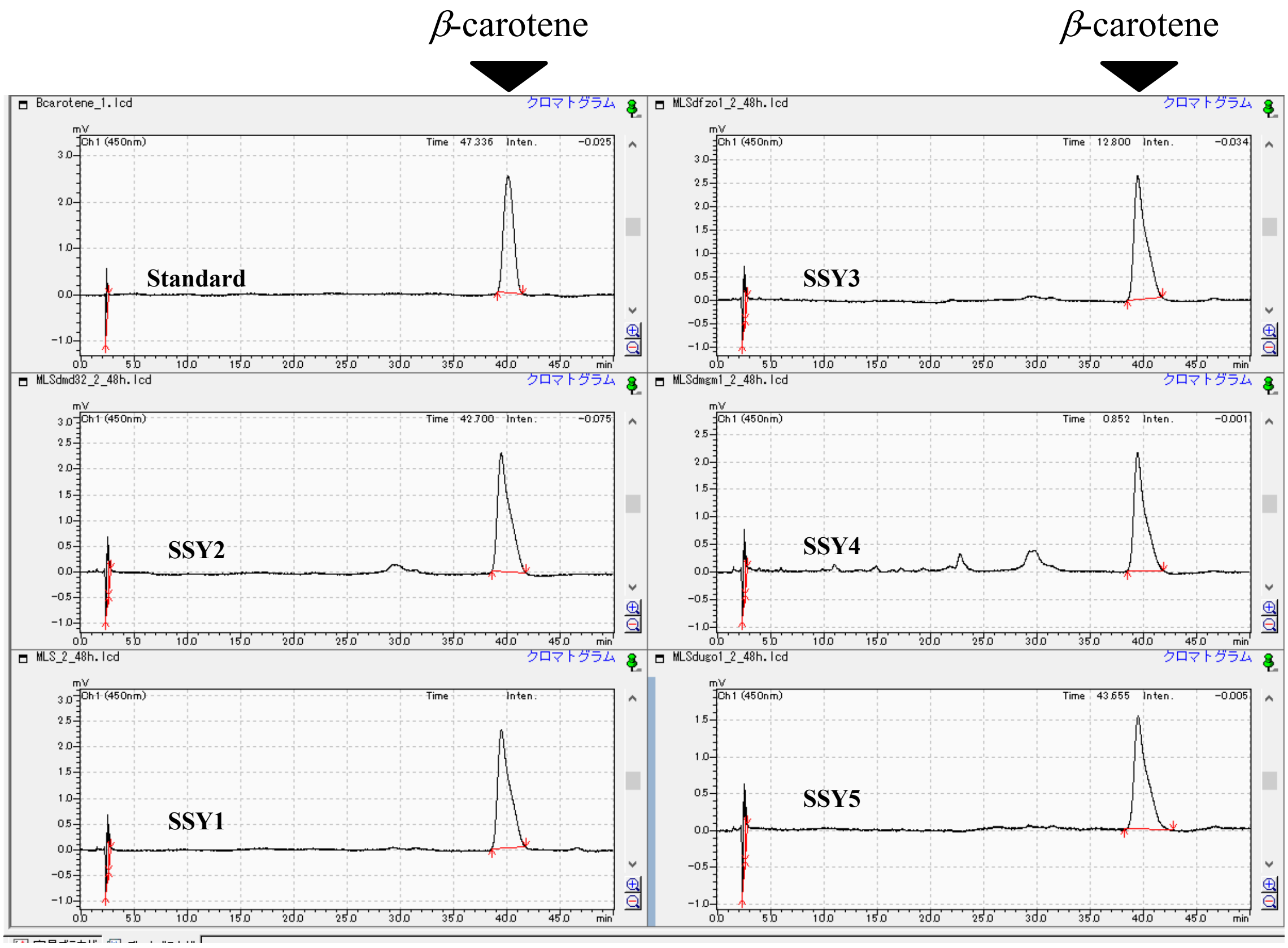

Fig. S3 The representative chromatograms of *β*-carotene

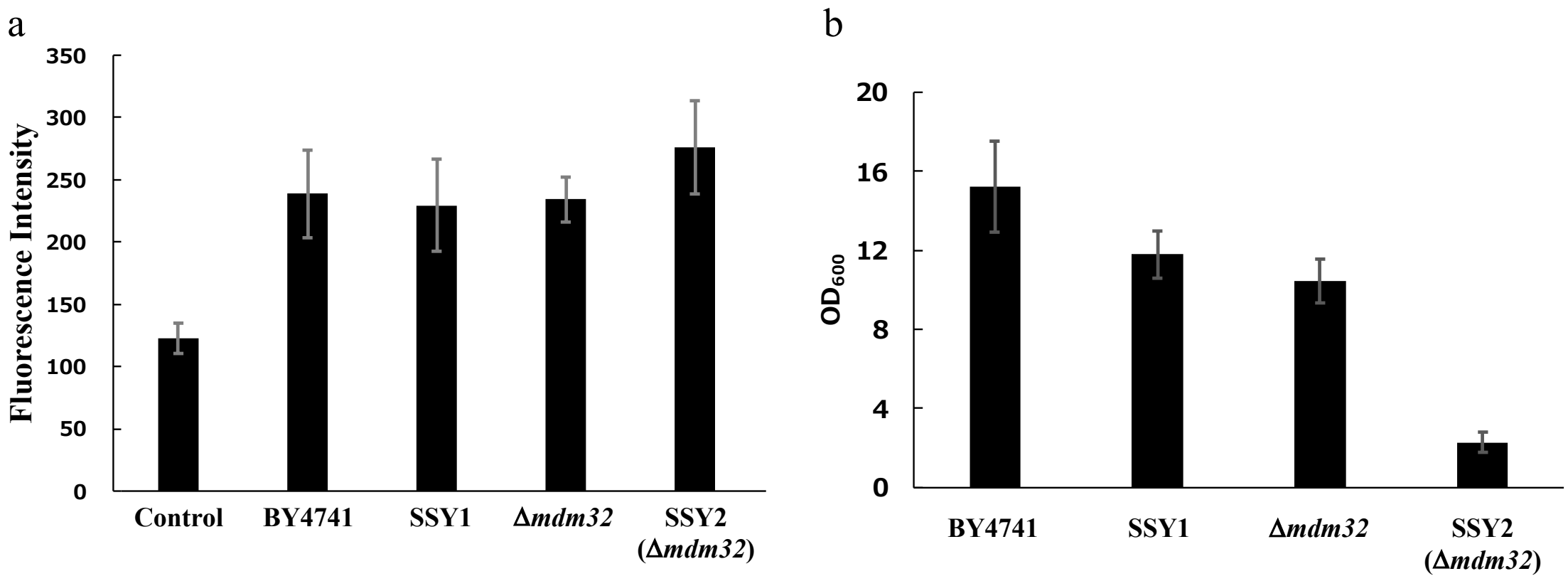

Fig. S4 Reactive oxygen species (ROS) level and growth of the strains

(a) ROS level and (b) growth of the BY4741, SSY1, Δ*mdm32*, and SSY2(Δ*mdm32*). Biological replication was achieved using three individual cells. Data are presented as the mean ± SD.

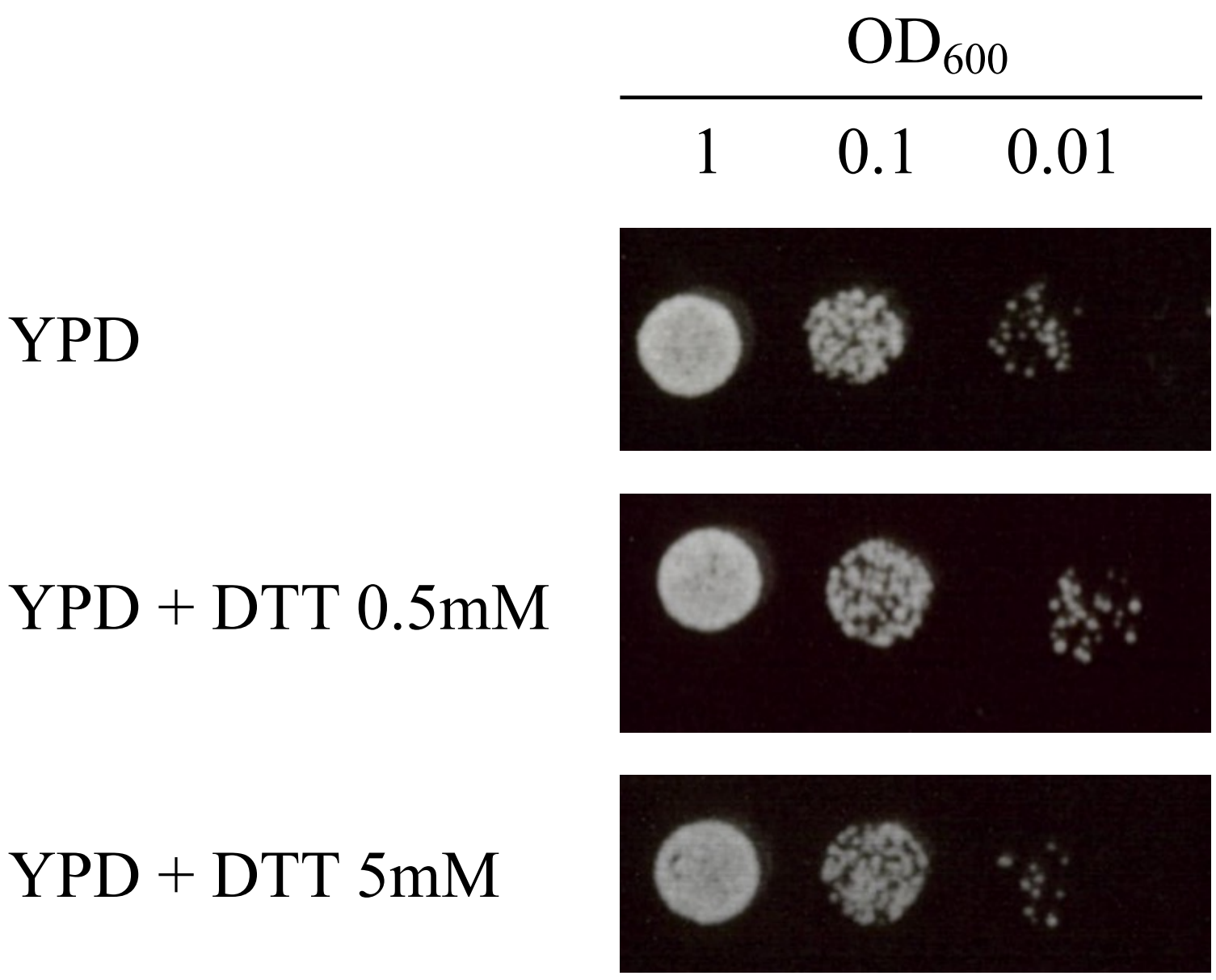

Fig. S5 The growth of SSY2 was not improved by DTT addition

Table S1 Plasmid list

Name	Description	Reference
pATP426	Expression vector containing PGK1,TDH3,ADH1 promoter, 2 μ origin and URA3 marker	Jun et al (2014)
pGK426	Expression vector containing PGK1 promoter, 2 μ origin and URA3 marker	Jun et al (2014)
pGK416-ymUkG1	ymUkG1 (yeast codon-optimized)	Kaishima et al (2016)
pGK426-MLS-GFP	pGK426, pPGK-MLS-GFP-tPGK	This study
pPreARS208	ΔARS208::tADH1-pADH1-pTDH3-tTDH3	This study
pPreARS308	ΔARS308::tADH1-pADH1-pTDH3-tTDH3	This study
pPreARS416	ΔARS416::tADH1-pADH1-pTDH3-tTDH3	This study
pARS208-*ERG13-ERG10*	ΔARS208::tADH1-*ERG13*-MLS-pADH1-pTDH3-MLS-*ERG10*-tTDH3	This study
pARS308-*ERG12-tHMG1*	ΔARS308::tADH1-*ERG12*-MLS-pADH1-pTDH3-MLS-*tHGM1*-tTDH3	This study
pARS416-*ERG19-ERG8*	ΔARS416::tADH1-*ERG19*-MLS-pADH1-pTDH3-MLS-*ERG8*-tTDH3	This study
pARS208-*ERG13-ERG10-URA*	ΔARS208::tADH1-*ERG13*-MLS-pADH1-*URA*-pTDH3-MLS-*ERG10*-tTDH3	This study
pARS308-*ERG12-tHMG1-URA*	ΔARS308::tADH1-*ERG12*-MLS-pADH1-*URA*-pTDH3-MLS-*tHGM1*-tTDH3	This study
pARS416-*ERG19-ERG8-URA*	ΔARS416::tADH1-*ERG19*-MLS-pADH1-*URA*-pTDH3-MLS-*ERG8*-tTDH3	This study
p*Fzo1*	Δ*fzo1*	This study
p*Mgm1*	Δ*mgm1*	This study
p*Ugo1*	Δ*ugo1*	This study
p*Mdm32*	Δ*mdm32*	This study
p*Fzo1-URA*	Δ*fzo1*::*URA*	This study
p*Mgm1-URA*	Δ*mgm1*::*URA*	This study

p*Ugo1*-*URA*	Δ*ugo1*::*URA*	This study
p*Mdm32*-*URA*	Δ*mdm32*::*URA*	This study
pRDH227	The plasmid carring hygromycin B resistance gene	Huang et al (2019)
pATP416-crtYBI	pATP416 vector with $crtYB_{Xd}$, $crtI_{Xd}$, and BTS1 cloned downstream of pADH1, pTDH3, and pPGK1, respectively	Masahiro et al (2021)
pCrtYBI-BTS1	The plasmid, in which the ura marker in pATP416-crtYBI was replaced to hygromycin B marker.	This study

Table S2 Primer list

ID	Group	Name	Sequence
1	pGK426-MLS-GFP_construction	MLS_UKG F1	TTTTCAAGCCAGCCACAAGAACTTTGTGTAGCTCTAGATATCTGCTTCAGATGGTCAGTGTCATCAAAGAAG
2	pGK426-MLS-GFP_construction	MLS_UKG F2	ACTAGTGGATCCCCCATGCTTTCACTACGTCAATCTATAAGATTTTTCAAGCCAGCCACAAGAAC
3	pGK426-MLS-GFP_construction	MLS_UKG R	GAATTCTCTAGACCCTTACTTAGAAGCTTGAGATGGC
4	pPreARS208_construction	ARS208_up_FW	TGATTACGCCAAGCTTTTAATTAAGGGTTCTGTTACTTGTCCA
5	pPreARS208_construction	ARS208_up_RV	GTCCTCAGAGGACAAGTGTGTATGGTCCCTATTG
6	pPreARS208_construction	Cassette_FW	TTGTCCTCTGAGGACATAAA
7	pPreARS208_construction	Cassette_RV	AAGTTTAGAAAATGCATCCTGGCGGAAAAAATTCA
8	pPreARS208_construction	ARS208_down_FW	GCATTTTCTAAACTTGTAGTTT
9	pPreARS208_construction	ARS208_down_RV	AGAGTGCACCATATGTTAATTAATAGCATTCTGGTTTGCCAAC
10	pPreARS308_construction	ARS308_up_FW	TGATTACGCCAAGCTTTTAATTAAGTTTTCGCCCATTGTTCTAG
11	pPreARS3	ARS308_up_	GTCCTCAGAGGACAATATATTCTGTTTGACAAGTGTT

	08_construction	RV	
12	pPreARS308_construction	ARS308_Cassette_RV	AAGAAGACAAGTAATATCCTGGCGGAAAAAATTCA
13	pPreARS308_construction	ARS308_down_FW	ATTACTTGTCTTCTTTGCTAC
14	pPreARS308_construction	ARS308_down_RV	TGAGAGTGCACCATATGTTAATTAAAAACTTTTTCAATCATTACCTTT
15	pPreARS416_construction	ARS416_up_FW	TGATTACGCCAAGCTTTTAATTAATTGGCTTTTTGATTGATTGTAC
16	pPreARS416_construction	ARS416_up_RV	GTCCTCAGAGGACAAAACGTGGGGTAAGTGCAC
17	pPreARS416_construction	ARS416_Cassette_RV	TCGGCACACAGTGGAATCCTGGCGGAAAAAATTCA
18	pPreARS416_construction	ARS416_down_FW	TCCACTGTGTGCCGAACA
19	pPreARS416_construction	ARS416_down_RV	TGAGAGTGCACCATATGTTAATTAACCATATCCACATCAATGGC
20	pARS208-*ERG13*-*ERG10*_construction	*ERG13*_FW	CGCGGCCGGCCGTTTTTATTTTTTAACATCGTAAGATCTTC
21	pARS208-*ERG13*-*ERG10*_construction	MLS_*ERG13*_RV1	AAAAAGTTCGGTCGGTGTTCTTGAAACACATCGAGATCTATAGACGAAGTCTACTTTGAGAGTTGATTTGAAA
22	pARS208-*ERG13*-	MLS_RV2	TCATATACACCTAGGATGCTTTCACTACGTCAATCTATAAGATTTTTCAAGCCAGCCACAAG

	*ERG10*_construction		
23	pARS208-*ERG13*-*ERG10*_construction	ADH1 promoter_FW	CCTAGGTGTATATGAGATAG
24	pARS208-*ERG13*-*ERG10*_construction	TDH3 promoter_MLS_RV	ACGTAGTGAAAGCATGTCGACTTTGTTTGTTTATGT
25	pARS208-*ERG13*-*ERG10*_construction	MLS_*ERG10*_FW1	TTTTCAAGCCAGCCACAAGAACTTTGTGTAGCTCTAGATATCTGCTTCAGATGTCTCAGAACGTTTACATT
26	pARS208-*ERG13*-*ERG10*_construction	MLS_FW2	ATGCTTTCACTACGTCAATCTATAAGATTTTCAAGCCAGCCACAAGAAC
27	pARS208-*ERG13*-*ERG10*_construction	*ERG10*_RV	ACGCGGCCGCACGCGTCATATCTTTTCAATGACAATAG
28	pARS308-*ERG12*-*tHMG1*_construction	*ERG12*_FW	CGCGGCCGGCCGTTTTTATGAAGTCCATGGTAAATTCG
29	pARS308-*ERG12*-*tHMG1*_construction	MLS_*ERG12*_RV1	TTTTTCAAGCCAGCCACAAGAACTTTGTGTAGCTCTAGATATCTGCTTCAGATGTCATTACCGTTCTTAACT
30	pARS308-*ERG12*-*tHMG1*_construction	MLS_*tHMG1*_FW1	TTTTCAAGCCAGCCACAAGAACTTTGTGTAGCTCTAGATATCTGCTTCAGATGGACCAATTGGTGAAAAC
31	pARS308-*ERG12*-	*tHMG1*_RV	ACGCGGCCGCACGCGTTAGGATTTAATGCAGGTGAC

	*tHMG1*_construction		
32	pARS416-*ERG19*-*ERG8*_construction	*ERG19*_FW	CGCGGCCGGCCGTTTTTATTCCTTTGGTAGACCAGTC
33	pARS416-*ERG19*-*ERG8*_construction	MLS_*ERG19*_RV1	TTTTTCAAGCCAGCCACAAGAACTTTGTGTAGCTCTAGATATCTGCTTCAGATGACCGTTTACACAGCATC
34	pARS416-*ERG19*-*ERG8*_construction	MLS_*ERG8*_FW1	TTTTCAAGCCAGCCACAAGAACTTTGTGTAGCTCTAGATATCTGCTTCAGATGTCAGAGTTGAGAGCCT
35	pARS416-*ERG19*-*ERG8*_construction	*ERG8*_RV	ACGCGGCCGCACGCGTTATTTATCAAGATAAGTTTCCG
36	pARS208-*ERG13*-*ERG10*-*URA*, pARS308-*ERG12*-*tHMG1*-*URA*, pARS416-*ERG19*-*ERG8*-*URA*_construction	*URA3*_upstream	ATATTGTACACCCCCTACCACAGCTTTTCAATTCAATTC
37	pARS208-*ERG13*-*ERG10*-*URA*, pARS308-	*URA3*_downstream	GTGTTTTTTATTCCCCGCATAGGGTAATAACTGAT

	ERG12-*tHMG1*-*URA*, pARS416-*ERG19*-*ERG8*-*URA*_construction		
38	*URA3*deletion_Donor	Promoter_ADH1side	TGTATATGAGATAGTTG
39	*URA3*deletion_Donor	Promoter_TDH3side	TTTGTTTGTTTATGTGTG
40	p*Fzo1*_construction	*Fzo1*_up_FW	TGATTACGCCAAGCTTTAATTAAAAGGGTCTCTTGACTGGGAG
41	p*Fzo1*_construction	*Fzo1*_up_down_RV	TGATTGCTGGCCATTTGTTGGCCACTGTTTTGGCATCTCT
42	p*Fzo1*_construction	*Fzo1*_down_Fw	AATGGCCAGCAATCAACCCA
43	p*Fzo1*_construction	*Fzo1*_down_RV	TGAGAGTGCACCATATTAATTAAATGTAGAATAACTACTAGGG
44	p*Mgm1*_construction	*Mgm1*_up_FW	TGATTACGCCAAGCTTTAATTAAGCGTAACACATGTGTCTTT
45	p*Mgm1*_construction	*Mgm1*_up_down_RV	AACCCATTAGAAAAATTCCTATGGAAACCGTTTTTTC
46	p*Mgm1*_construction	*Mgm1*_down_Fw	TTTTTCTAATGGGTTGTATC
47	p*Mgm1*_construction	*Mgm1*_down_RV	TGAGAGTGCACCATATTAATTAAGGTTCATATATGCAAAAGC
48	p*Ugo1*_construction	*Ugo1*_up_FW	TGATTACGCCAAGCTTTAATTAAGATTTAGCTTTTTGTTCCC
49	p*Ugo1*_construction	*Ugo1*_up_down_RV	ACAAATTCAGTCATTGCCTAGATTGCGCGGAGGG
50	p*Ugo1*_construction	*Ugo1*_down_Fw	AATGACTGAATTTGTGCTACT
51	p*Ugo1*_construction	*Ugo1*_down_RV	TGAGAGTGCACCATATTAATTAACAGAAAGACAGCAAATA

52	p*Mdm32*_construction	*Mdm32*_up_pCU19_FW	TGATTACGCCAAGCTTTAATTAACCCCCATCTTGTCCACTTACAGG
53	p*Mdm32*_construction	*Mdm32*_up_down_RV	TATAAGCAATAGTCACATGAATTTTATAAACCTCCC
54	p*Mdm32*_construction	*Mdm32*_down_FW	TGACTATTGCTTATATAATATC
55	p*Mdm32*_construction	*Mdm32*_down_pCU19_RV	TGAGAGTGCACCATATTAATTAAAGTGAAAATGCGGATACC
56	p*Fzo1*-*URA*_construction	*Fzo1*_up_URA3_RV	ATGAATTGAATTGAAAAGCTCCCGGGTGTTGGCCACTGTTTTGGC
57	p*Fzo1*-*URA*_construction	*URA3*_FW	AGCTTTTCAATTCAATTCATCAT
58	p*Fzo1*-*URA*_construction	*URA3*_RV	GGGTAATAACTGATATAATTAAATTGAAGC
59	p*Fzo1*-*URA*_construction	*Fzo1*_down_*URA3*_FW	TATCAGTTATTACCCAATGGCCAGCAATCAACC
60	p*Mgm1*-URA_construction	*Mgm1*_up_RV	ATGAATTGAATTGAAAAGCTCCCGGGTTCCTATGGAAACCGTTTTTTCAAGG
61	p*Mgm1*-URA_construction	*Mgm1*_down_FW	TATCAGTTATTACCCTTTTTCTAATGGGTTGTATC
62	p*Ugo1*-URA_construction	*Ugo1*_up_RV	TTGAATTGAAAAGCTCCCGGGGCCTAGATTGCGCGGAG
63	p*Ugo1*-URA_construction	*Ugo1*_down_FW	TATCAGTTATTACCCAATGACTGAATTTGTGCTAC
64	p*Mdm32*-URA_construction	*Mdm32*_up_*URA3*_RV	TTGAATTGAAAAGCTCATGAATTTTATAAACCTCCC
65	p*Mdm32*-	*Mdm32*_down	TATCAGTTATTACCCTGACTATTGCTTATATAATATC

	URA_construction	_*URA3*_FW	
66	*Fzo1*_DonorDNA	*Fzo1*_up_FW_Donor	AAGGGTCTCTTGACTGGGAGTTC
67	*Fzo1*_DonorDNA	*Fzo1*_down_RV_Donor	ATGTAGAATAACTACTAGGGG
68	*Mgm1*_DonorDNA	*Mgm1*_up_FW_Donor	GCGTAACACATGTGTCTTTT
69	*Mgm1*_DonorDNA	*Mgm1*_down_RV_Donor	GGTTCATATATGCAAAAGCAG
70	*Ugo1*_DonorDNA	*Ugo1*_up_FW_Donor	GATTTAGCTTTTTGTTCCCT
71	*Ugo1*_DonorDNA	*Ugo1*_down_RV_Donor	CAGAAAGACAGCAAATATAA
72	*Mdm32*_DonorDNA	*Mdm32*_up_FW_Donor	CCCCCATCTTGTCCACTTACAGG
73	*Mdm32*_DonorDNA	*Mdm32*_down_RV_Donor	AGTGAAAATGCGGATACC
74	pCrtYBI-BTS1_construction	*HygB_up*	AACTGCACAGAACAAAAACCTGCAGGGACATGGAGGCCCAGAATACC
75	pCrtYBI-BTS1_construction	*HygB_down*	ATTTGTGAGTTTAGTATACATGCATCAGTATAGCGACCAGCATTCACA

Table S3 The DNA fragments list

Primers		Template	DNA fragment
Forward	Reverse		
1	3	pGK416-ymUkG1	1
2	3	DNA fragment 1	2
4	5	*S. cerevisiae* Genome	3
6	7	pATP426	4
8	9	*S. cerevisiae* Genome	5
20	21	*S. cerevisiae* Genome	6
20	22	DNA fragment 6	7
25	27	*S. cerevisiae* Genome	8
26	27	DNA fragment 8	9
23	24	pATP426	10
36	37	pGK426	11
10	11	*S. cerevisiae* Genome	12
6	12	pATP426	13
13	14	*S. cerevisiae* Genome	14
28	29	*S. cerevisiae* Genome	15
28	22	DNA fragment 15	16
30	31	*S. cerevisiae* Genome	17
26	31	DNA fragment 17	18
15	16	*S. cerevisiae* Genome	19
6	17	pATP426	20
18	19	*S. cerevisiae* Genome	21
32	33	*S. cerevisiae* Genome	22
32	22	DNA fragment 22	23
34	35	*S. cerevisiae* Genome	24
26	35	DNA fragment 24	25
40	41	*S. cerevisiae* Genome	26
42	43	*S. cerevisiae* Genome	27
40	56	*S. cerevisiae* Genome	28
57	58	pGK426	29

59	43	*S. cerevisiae* Genome	30
44	45	*S. cerevisiae* Genome	31
46	47	*S. cerevisiae* Genome	32
44	60	*S. cerevisiae* Genome	33
61	47	*S. cerevisiae* Genome	34
48	49	*S. cerevisiae* Genome	35
50	51	*S. cerevisiae* Genome	36
48	62	*S. cerevisiae* Genome	37
63	51	*S. cerevisiae* Genome	38
52	53	*S. cerevisiae* Genome	39
54	55	*S. cerevisiae* Genome	40
52	64	*S. cerevisiae* Genome	41
65	55	*S. cerevisiae* Genome	42
38	39	pATP426	43
66	67	p*Fzo1-URA3*	44
66	67	p*Fzo1*	45
68	69	p*Mgm1-URA3*	46
68	69	p*Mgm1*	47
70	71	p*Ugo1-URA3*	48
70	71	p*Ugo1*	49
72	73	p*Mdm32-URA3*	50
72	73	p*Mdm32*	51
74	75	pRDH227	52
